# Modulation of spinal excitability following neuromuscular electrical stimulation superimposed to voluntary contraction

**DOI:** 10.1007/s00421-020-04430-5

**Published:** 2020-07-17

**Authors:** Riccardo Borzuola, Luciana Labanca, Andrea Macaluso, Luca Laudani

**Affiliations:** 1 Department of Movement, Human and Health Sciences, University of Rome “Foro Italico”, Rome, Italy; 2 Cardiff School of Sport and Health Sciences, Cardiff Metropolitan University, Cardiff, UK

**Keywords:** NMES, H-reflex, Soleus, Triceps surae, Motor unit recruitment

## Abstract

**Purpose:**

Neuromuscular electrical stimulation (NMES) superimposed on voluntary muscle contraction has been recently shown as an innovative training modality within sport and rehabilitation, but its effects on the neuromuscular system are still unclear. The aim of this study was to investigate acute responses in spinal excitability, as measured by the Hoffmann (H) reflex, and in maximal voluntary contraction (MVIC) following NMES superimposed to voluntary isometric contractions (NMES + ISO) compared to passive NMES only and to voluntary isometric contractions only (ISO).

**Method:**

Fifteen young adults were required to maintain an ankle plantar-flexor torque of 20% MVC for 20 repetitions during each experimental condition (NMES + ISO, NMES and ISO). Surface electromyography was used to record peak-to-peak H-reflex and motor waves following percutaneous stimulation of the posterior tibial nerve in the dominant limb. An isokinetic dynamometer was used to assess maximal voluntary contraction output of the ankle plantar flexor muscles.

**Results:**

H-reflex amplitude was increased by 4.5% after the NMES + ISO condition (*p* < 0.05), while passive NMES and ISO conditions showed a decrease by 7.8% (*p* < 0.05) and no change in reflex responses, respectively. There was no change in amplitude of maximal motor wave and in MVIC torque during each experimental condition.

**Conclusion:**

The reported facilitation of spinal excitability following NMES + ISO could be due to a combination of greater motor neuronal and corticospinal excitability, thus suggesting that NMES superimposed onto isometric voluntary contractions may provide a more effective neuromuscular stimulus and, hence, training modality compared to NMES alone.

## Introduction

Neuromuscular electrical stimulation (NMES) consists of intermittent electrical stimuli applied to one or more superficial skeletal muscles (Botter et al. 2011) to preserve muscle mass and contractile function in either healthy or injured individuals (Gibson et al. 1988; Caggiano et al. 1994; Bax et al. 2005; Sheffler and Chae 2007; Vanderthommen and Duchateau 2007). To date, the neurophysiological mechanisms underpinning motor unit recruitment induced by NMES are still unclear, with some authors suggesting that NMES leads to spatially fixed (Vanderthommen et al. 2000), non-selective (Adams et al. 1993) and largely incomplete motor unit recruitment (Vanderthommen et al. 2003; Gregory and Bickel 2005). In particular, it has been shown that NMES induced motor unit recruitment at low intensity involves initially large high-threshold motor units, which are constituted of type II fast-twitch fibres generally located in the superficial regions of the skeletal muscles (Bickel et al. 2011). On the other hand, deeper regions of the muscle could be reached by progressively increasing the intensity of the stimulation, leading to a progressive depolarization of other populations of muscle fibres (Maffiuletti 2010). It is important to note that this recruitment order is opposed to the recruitment pattern that is normally observed during voluntary contractions (Henneman 1957), which involves first an asynchronous recruitment of small, type I slow-twitch fatigue resistant muscle fibres and then it is followed by a recruitment of increasingly larger, type II fast-twitch fibres.

Interesting findings have been reported by delivering NMES while voluntarily performing movement exercises (NMES+), which was shown to improve motor performance in both healthy and pathological individuals to a greater extent than NMES only or voluntary exercise training alone (Paillard et al. 2005; Wahl et al. 2012, 2015; Labanca et al. 2018; Paillard 2018). Noteworthy, Labanca et al. (2018) revealed that NMES+ applied over the muscle belly is effective to safely regaining quadriceps strength after knee surgery, particularly in injured individuals whose voluntary activation capacities are impaired or temporarily disrupted. Results from previous neurophysiological studies suggest that a plausible reason underpinning the effectiveness of NMES+ superimposed to voluntary movement might involve an increase in spinal excitability due to plastic changes in Ia reflex pathways, which could allow for a more comprehensive motor unit recruitment and force-generating capabilities compared to NMES alone and/or voluntary movement alone.

The Hoffman reflex (H-reflex) technique has been adopted in several previous studies to investigate the acute effects of either NMES+, voluntary contractions or NMES alone on the spinal reflex responses, with interesting findings. Unaltered (Lagerquist et al. 2012) or suppressed (Milosevic et al. 2019) H-reflex responses were reported after a single set of isometric voluntary contractions. A considerable H-reflex attenuation was observed immediately following passive NMES (Lagerquist et al. 2012; Papaiordanidou et al. 2014; Wegrzyk et al. 2015; Gueugneau et al. 2017; Grosprêtre et al. 2018). On the other hand, Lagerquist et al. (2012) reported increased H-reflex responses after a single session of NMES+ obtained by electrical stimulation of the tibial nerve superimposed with voluntary contraction of the ankle plantar-flexor muscles, while H-reflexes did not change after performing either voluntary contraction or nerve stimulation alone. Interestingly, it was suggested that the increased spinal excitability could allow for greater force production when the nerve electrical stimulation was superimposed to the voluntary contraction compared to either voluntary contraction or nerve stimulation. It is not known, however, whether NMES+ applied over the muscle could lead to increased spinal reflex response similarly to what was reported following direct nerve stimulation superimposed to voluntary contraction. Yet, it should be noted that muscle contractions evoked by direct nerve stimulation are less stable and unable to produce large torques compared to percutaneous NMES applied over the muscle (Baldwin et al. 2006; Klakowicz et al. 2006), which could explain why the latter has emerged as a preferable option to be integrated in training protocols.

Therefore, the aim of this study was to investigate the acute response of the H-reflex following NMES applied on the triceps surae muscle while performing voluntary isometric contractions (NMES + ISO) compared to muscle electrical stimulation only (NMES) and voluntary isometric contractions only (ISO). Based on the previous findings by Lagerquist et al. (2012) reporting increased spinal excitability after voluntary muscle contractions combined with electrical nerve stimulation, it was hypothesized that the soleus H-reflex response would be greater immediately after NMES + ISO compared to either NMES or ISO. The soleus muscle was investigated in this study because amplitude of its H-reflex is typically greater and more stable with respect to other skeletal muscles (Táboríková and Sax 1968; Zehr 2002), allowing for a reliable evaluation of the reflex responses and their modulation.

## Materials and methods

### Participants

Fifteen young healthy volunteers (eight males and seven females, mean ± SD age: 26 ± 4 years, mass: 69 ± 11 kg, height: 1.73 ± 0.11 m), with no history of neurological or orthopaedic disorders, volunteered to participate in the study. The sample size was determined a priori based on a statistical power analysis (G*Power software version 3.1.9.4; *α* = 0.05, power = 0.95, effect size = 0.45) for repeated-measure ANOVA, according to Cohen (1992). Individuals who were physically active but did not engage in sport practice more than three times a week, for more than 60 min each time, were included in the study. None of the participants had experience with NMES exercise before performing the experimental session. The study was approved by the institutional ethics review board and all participants gave informed written consent before participating.

### Experimental design

**Fig. 1:**
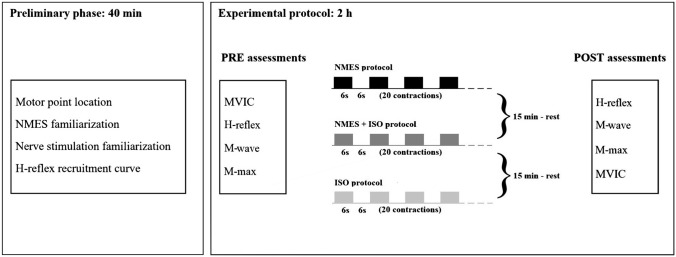
Diagram of the experimental protocol. *NMES* neuromuscular electrical stimulation only, *NMES + ISO* neuromuscular stimulation superimposed on voluntary isometric contraction, *ISO* isometric voluntary contraction, *test H-reflex* test H-reflex before exercise protocol, *test M-wave* small M-wave corresponding to test H-reflex, *M-max* maximal M-wave, *MVIC* maximal voluntary isometric contraction, *H-reflex* test H-reflex after exercise protocol

A single group, repeated measures study design was adopted for this study with three experimental conditions: (a) NMES applied on the triceps surae muscle (NMES), (b) NMES superimposed on voluntary isometric contraction of the ankle plantar flexor muscles (NMES + ISO) and c) voluntary isometric contraction of the ankle plantar flexor muscles only (ISO). Conditions were administered in a random order to each participant during a unique experimental session, which lasted between 2.5 and 3 h. Each condition involved 20 intermittent contractions (6 s contraction/6 s rest) for a total duration of 4 min. Participants were allowed a 15 min period of recovery between conditions. This protocol, including the number and duration of contractions, was chosen in order to prevent development of muscle fatigue while at the same time modulating spinal excitability, based on previous studies’ reports (Neyroud et al. 2014; Grosprêtre et al. 2018). All the procedures were performed on each participant’s dominant leg as described by Botter et al. (2011). Leg dominance was determined as the limb preferred for hopping or kicking a ball (Holmbäck et al. 2003). Before and after each condition, a set of neuromechanical assessments was carried out, with simultaneous recording of surface electromyography (sEMG) as shown in Fig. 1. First, participants completed a maximal voluntary isometric contraction (MVIC) assessment and an assessment of the Hoffman reflex (H-reflex) recruitment curve. Each condition was preceded by assessments of a baseline H-reflex and maximal motor wave (M-max) and immediately followed by a post-treatment H-reflex assessment, an ulterior M-max and a repetition of the MVIC.

### Electromyographic recordings

**Fig. 2:**
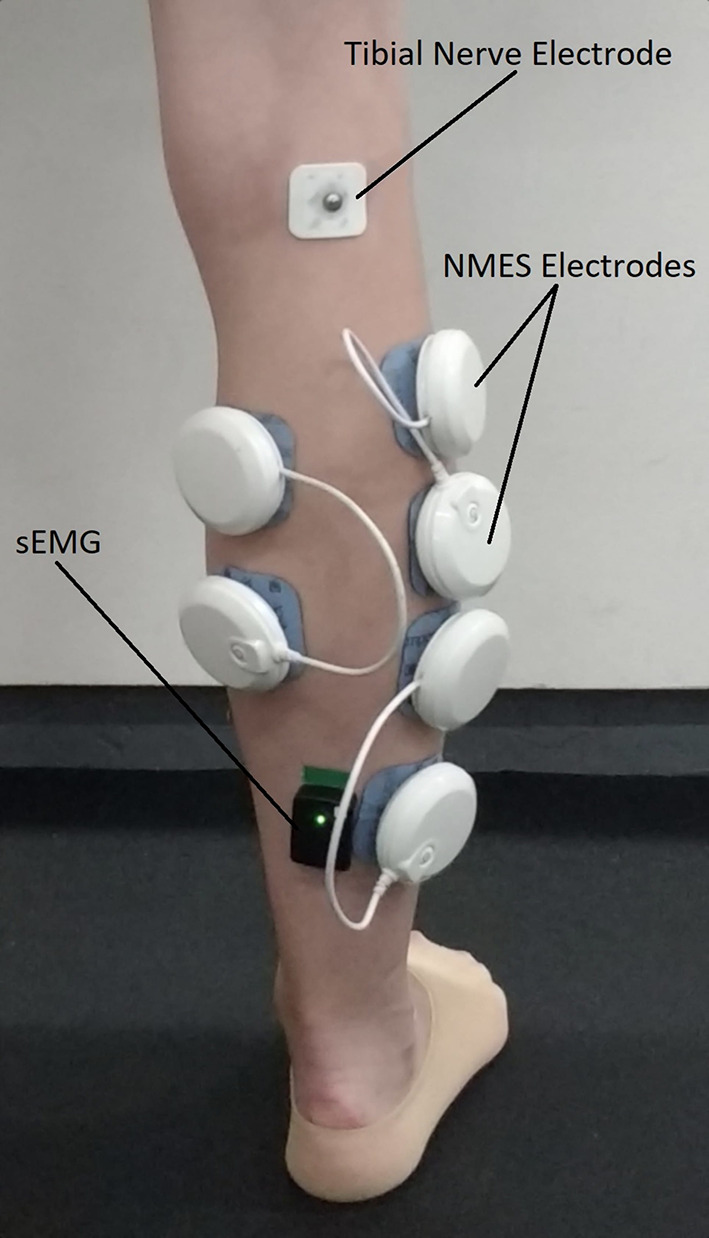
Electrodes (recording and stimulating) positioning. White pods represent the neuromuscular stimulator active wireless electrodes placed in a bipolar configuration on the soleus, gastrocnemius lateralis and gastrocnemius medialis muscles. *sEMG* surface electromyography of the soleus muscle

Surface electromyography (sEMG) was recorded by means of a wireless system (Delsys Trigno, Boston, MA, USA) at a sample rate of 2000 Hz. Surface electrodes were placed on the soleus (SOL) muscle 2–3 cm below the gastrocnemii musculotendinous junction with Achille’s tendon (Fig. 2), and on the tibialis anterior (TA) muscle above the muscle belly parallel to the TA muscle fibres. TA muscle was chosen to control any possible pre-activation of the antagonist muscles during H-reflex assessments, which is known to significantly affect H-reflex responses of the soleus (Hoffmann 1952). Before applying the surface electrodes, participants’ skin was shaved and gently abraded with an abrasive paste to keep impedance below 5 kΩ and thus promote electrical signal transmission.

### MVIC

An isokinetic dynamometer (Cybex Humac NORM II, Cybex, New York, US) was used to assess MVIC of the ankle plantar flexor muscles. Participants were positioned with the hip at 90° (0° = neutral hip position), the knee at 60° (0° = full knee extension) and the ankle at 0° of ankle plantar-dorsi flexion (0° = foot orthogonal to the shank axis) with the lateral malleolus aligned with the axis of the dynamometer, the foot firmly secured to the device footplate and their trunk and knee fastened by instrumented belts. Following warm-up and familiarisation during which participants performed 15–20 submaximal isometric contractions, the MVIC assessment consisted of a quick increase to a maximum in the force exerted by the plantar flexor muscles. Participants had a visual feedback of their performance on a computer screen and were verbally encouraged to promote their maximal isometric contraction and maintain it for at least 3 s before relaxing. A minimum of 3 attempts were performed, with each attempt being separated by 3 min rest intervals to minimize fatigue. MVIC was chosen as the largest 1-s average achieved within a torque recording. Assessment of MVIC was then used to define a target isometric plantarflexion torque as 20% of MVIC, which represents the constant torque that participants were required to achieve during the three experimental conditions.

### NMES

A muscle stimulator (Chattanooga Wireless Professional, DJO Global, Vista, CA, USA) was used to deliver NMES over the triceps surae muscles to evoke low intensity muscle contractions either passively or superimposed on voluntary effort. The stimulator produced a rectangular, balanced biphasic pulse and was always safely handled and controlled by the investigator. Self-adhesive electrodes (Compex Dura-Stick plus) with positive polarity were placed over the motor points of gastrocnemius lateralis, gastrocnemius medialis, and soleus muscles (Fig. 2). Motor points were identified at the beginning of the experimental session with a hand-held cathode ball electrode in accordance with the electrical stimulator user’s guide. In addition, three self-adhesive electrodes with negative polarity were placed on each muscle about 3 cm above the positive electrodes located on the motor points. NMES was delivered with a pulse frequency of 50 Hz and a pulse width of 400 μs to effectively stimulate the triceps surae while at the same time promoting the highest comfort during stimulation as reported in previous investigation (Maffiuletti 2010). The current pulse intensity of the stimulation was manually adjusted in accordance to each participant’s tolerance. Before the beginning of the experimental conditions participants familiarized with the electrical stimuli for a few minutes. NMES was immediately suspended if participants showed any signs of pain or discomfort. During NMES, current pulse intensity was increased until the passively stimulated plantarflexion produced the torque target at 20% of MVIC. During ISO, participants were required to match the torque target of 20% of MVIC by voluntarily contracting their plantar flexor muscles. During NMES + ISO, current pulse intensity was set to generate half of the torque target (10% of MVIC) and participants were asked to voluntarily contract their plantar-flexor muscles to achieve the full torque target of 20% of MVIC. Participants were asked to relax their calf muscles before the first and after the tenth contraction while the experimenter adjusted the pulse intensity to ensure that the torque produced corresponded to half of the torque target throughout the NMES + ISO condition.

### Reflex and motor recordings

**Fig. 3:**
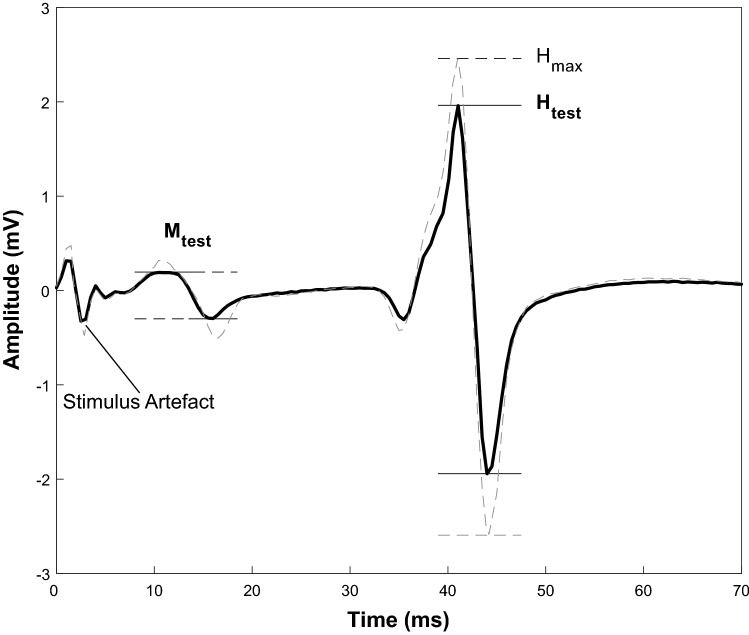
Test H-reflex selection. Test H-reflex sEMG data (black solid line) with the small m-wave (test M-wave) and the corresponding test H-reflex (80–85% of H-max). Maximal H-reflex sEMG data (grey dashed line) with corresponding H-max

Single rectangular biphasic pulses, with a duration of 1 ms were delivered to the tibial nerve via a constant voltage electrical stimulator (Digitimer DS7A, Hertfordshire, AL7 3BE, England, UK). The optimal stimulation site was located using a hand-held cathode ball electrode in the popliteal fossa. A self-adhesive cathode was placed in the selected stimulation site and firmly secured with medical adhesive tape while the anode was secured anteriorly on the knee above the patella. The soleus H-reflex recruitment curve was then obtained according to previously adopted procedures (Zehr 2002). Low intensity single electrical stimuli between 1 and 99 mA were delivered to the tibial nerve every 10 s to avoid development of muscle fatigue. Stimulus intensity was increased at steps of 1 mA from motor threshold (smallest H-reflex) to maximal M-wave (M-max). Each stimulus induced an involuntary contraction of the triceps surae muscle, which was recorded via sEMG and visually monitored by the investigator immediately after the stimulus. Peak-to-peak analysis of the sEMG trace was used to define the amplitude of H-reflexes and M-waves. The test reflex stimulus intensity was determined to obtain an H-reflex on the ascending limb of the recruitment curve with a peak-to-peak amplitude lying between 80 and 85% of the maximal H-reflex (H-max), as detailed in previous research (Zehr 2002; Lagerquist et al. 2006). A small M-wave (test M-wave), corresponding to the test H-reflex, was selected and monitored throughout the entire experiment in order to control the stimulus consistency and repeatability before and after each condition (Fig. 3). If the evoked H-reflexes showed an M-wave with an amplitude within a range of ± 5% of the selected test M-wave, the measure was accepted and kept for further analysis. We collected a minimum of five accepted H-reflex during each neuromechanical assessment before and after each condition. Two M-max responses were also recorded after the test H-reflexes. All H-reflex and M-wave amplitudes were normalized to the M-max amplitude, averaged and used for off-line analysis.

### Data analysis and statistics

All data were analysed using a custom Matlab code (Matlab 2015b, Mathworks Inc., Natick, MA, USA). For each stimulation, sEMG traces of TA and SOL were checked to determine if any pre-activation had occurred before the reflex measure. sEMG traces which revealed pre-activation of either SOL or TA were removed from data analysis and thus its corresponding H-reflex measure. Statistical analysis was performed using IBM SPSS 24.0 (IBM Corp., Armonk, NY, USA). A two-way repeated measures analysis of variance (ANOVA) was used to evaluate statistical difference in H-reflex, M-wave and MVIC measures between exercise conditions (NMES, NMES + ISO, ISO) and over time (PRE, POST) as within-subjects factors. The Mauchly test was used to check the data for normality and sphericity. When a significant interaction between exercise condition and time was found, a repeated-measures ANOVA was performed to detect significant differences between PRE and POST measures within each exercise condition. A Bonferroni correction was applied with and level of significance set at 0.05.

## Results

**Fig. 4:**
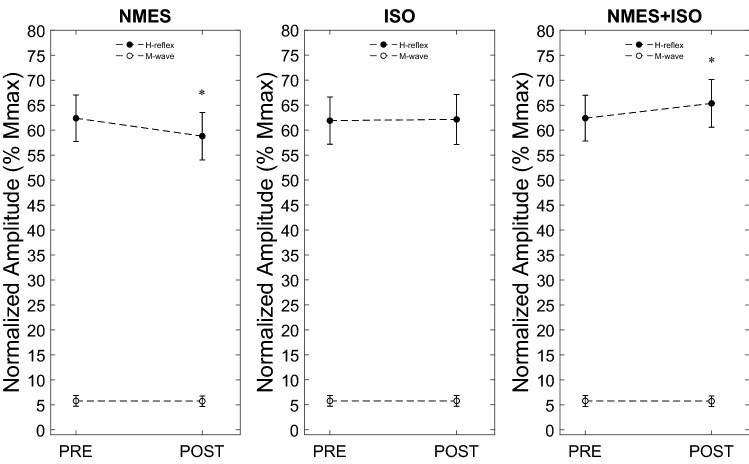
Amplitude of the Soleus H-reflex and associated M-waves normalised to M-max before (PRE) and after (POST) passive NMES applied to the triceps surae muscle (NMES), NMES superimposed on voluntary isometric contraction of the plantar flexor muscles (NMES + ISO), and isometric voluntary contraction of the plantar flexor muscles alone (ISO). Data are showed as group means ± standard deviation. *Significant difference between PRE and POST (*p* < 0.05)

The repeated measures ANOVA showed a main effect of Time (*F* = 785.712, *η*_p_^2^ = 0.982, *p* < 0.001), Condition (*F* = 993.454, *η*_p_^2^ = 0.986, *p* < 0.001), and a time × condition interaction (*F* = 909.820, *η*_p_^2^ = 0.985, *p* < 0.001) on the normalized H-reflex amplitude. Further analysis indicated a significant effect of time on the normalized H-reflex amplitude for the NMES (*F* = 4.879, *η*_p_^2^ = 0.258, *p* < 0.05) and NMES + ISO (*F* = 6.526, *η*_p_^2^ = 0.318, *p* < 0.05) conditions. As shown in Fig. 4, H-reflex amplitude decreased on average by 7.8% following passive NMES compared to baseline. On the contrary_,_ H-reflex amplitude significantly increased on average by 4.5% in the NMES + ISO condition compared to baseline.

There was no concomitant difference in the normalized test M-wave corresponding to the test H-reflexes. Indeed, statistical analysis showed no time × condition interaction (*p* > 0.05).

We also found no significant difference in the amplitude of M-max across condition and time as shown in Table 1. Results showed no time × condition interaction (*p* > 0.05).

**Table 1:** Maximal M-waves (M-max) of the soleus muscle before (PRE) and after (POST) each condition

M-max (mV)	NMES	NMES + ISO	ISO
PRE	4.12 ± 1.15	4.11 ± 1.15	4.11 ± 1.17
POST	4.12 ± 1.16	4.12 ± 1.16	4.17 ± 1.17

Data are presented as group means ± standard deviation

As shown in Table 2, the ANOVA showed no significant main effect or interaction of time and condition for MVIC (*p* > 0.05).

**Table 2:** Maximal isometric voluntary contraction (MVIC) of the ankle plantar flexor muscles measured before the beginning of the experimental conditions and after each condition

	Pre-test	NMES	NMES + ISO	ISO
MVIC (Nm)	43.80 ± 16.99	43.82 ± 16.01	43.15 ± 16.22	43.79 ± 14.26

Data are presented as group means ± standard deviation

## Discussion

The main finding of this study was that NMES superimposed on isometric voluntary contraction of the ankle plantar flexor muscles (NMES + ISO) induced acute potentiation of the soleus H-reflex amplitude compared to baseline values, whereas the H-reflex was inhibited after passive NMES only and did not change after the isometric voluntary contractions only.

The increase in H-reflex amplitude after the current NMES + ISO exercise could reflect a specific spinal modulation, e.g. increased excitability of the stretch reflex pathway (Zehr 2002), particularly involving the smaller soma size motoneurons innervating low-threshold type I motor fibres that are located in the deepest region of the soleus muscle (Edström and Nyström 1969; Gollnick et al. 1974). Neural adaptations affecting the H-reflex gain could involve either improved descending facilitation of the motoneuron excitability or modulation of presynaptic and postsynaptic facilitation/inhibition (Zehr 2002). Increased cortical excitability has been reported when voluntary contractions were paired with NMES (Khaslavskaia and Sinkjaer 2005; Barsi et al. 2008), thus indicating that neuroplastic changes at the cortical level could have been partly responsible for the H-reflex potentiation after the NMES + ISO condition in our study. On the other hand, Lagerquist et al. (2012) showed that a combination of tibial nerve stimulation and voluntary contractions of the ankle plantar flexors potentiated spinal excitability but did not alter cortical excitability, suggesting that presynaptic mechanisms are predominantly involved in the H-reflex modulation, whereas cortical and postsynaptic mechanisms appear to not significantly contribute (Lagerquist et al. 2012). Other authors have argued that a lack in cortical excitability alterations could be due to the scarce supraspinal control on the soleus muscle compared to other skeletal muscles (Brooke et al. 1997; Maertens De Noordhout et al. 1999; Bawa et al. 2002).

In our study, H-reflex amplitude was unaltered after the ISO condition. This is in agreement with Lagerquist et al. (2012) who reported no changes in H-reflex after a 40 min intermittent exercise protocol consisting of isometric voluntary contractions of the ankle plantar flexors at sub-maximal torque level. On the contrary, Milosevic et al. (2019) showed acute suppression of the H-reflex immediately after a 60 s continuous isometric ankle plantar-flexion exercise. This discrepancy with our study results could be due to differences in the voluntary exercise protocol, as the continuous protocol adopted by Milosevic et al (2019) may have increased the inhibitory discharge of afferent pathways, likely from the muscle spindles (Masugi et al. 2017).

The reduction in H-reflex magnitude after the NMES condition is in agreement with previous studies’ findings (Lagerquist et al. 2012; Papaiordanidou et al. 2014; Wegrzyk et al. 2015; Gueugneau et al. 2017; Grosprêtre et al. 2018) and might reflect decreased spinal excitability as a result of reduced input from type Ia afferences to the α-motoneurons. Martin et al. (2016) argued that repetitive stimulation of the Ia afferent may induce a hyperpolarization of the axonal branches which would increase their excitability threshold and diminish their response to the same stimulus intensity. In the study of Grosprêtre et al. (2018), it was found that decline in spinal excitability of the soleus muscle was caused by an increase in presynaptic inhibitory adjustments including homosynaptic postactivation depression of the Ia terminals and, even to a higher extent, primary afferent depolarization. A similar attenuation of the soleus H-reflex was reported during protocols involving external mechanical stimuli such as local or whole-body vibration (WBV). In line with that, a recent study of Laudani et al. (2018) indicated that one minute of non-fatiguing WBV exercise lead to an acute decrease of the soleus H-reflex response; similar to the interpretation proposed by Grospêtre et al. for electrical stimuli (2018), it was suggested that repetitive activation of the Ia-motoneuron synapse in response to vibratory stimuli may induce inhibitory mechanisms including primary afferent depolarization and post-activation depression which are, therefore, expressed by the H-reflex attenuation (Pierrot-Deseilligny and Mazevet 2000). Another finding of the current study was that neither M-max or MVIC differed following NMES+, NMES only and ISO conditions, which would support the hypothesis that neural adaptations are presumably related to corticospinal rather than peripheral mechanisms, such as change in muscle membrane excitability (Wegrzyk et al. 2015).

There were a few potential limitations in our study. First, we recruited healthy active participants with potentially different training/activity backgrounds. This training-type heterogeneity possibly affected the H-reflex results due to the different reflex sensitivity of participants performing either explosive or endurance activity. For instance, previous studies’ results have suggested that explosive-type athletes, given the higher proportion of high-threshold motor units, would exhibit decreased H-reflex responses (Casabona et al. 1990), especially on the ascending limb of the recruitment curve (Maffiuletti et al. 2001). Moreover, Pérot et al. (1991) reported an increase in the H-reflex responses after several weeks of endurance training, indicating higher reflex sensitivity and motoneuron excitability in participants who performed this type of activity. Second, it could be argued that our testing protocol of 4 min duration involving 20 isometric contractions at 20% of the MVIC might have not been sufficient to induce significant corticospinal adaptations. With this regard, we aimed at analysing the acute H-reflex response after a non-fatiguing intervention and our MVIC results confirm that muscle fatigue did not arise following any of the three experimental conditions. As previously stated, the majority of the previous studies using longer intervention protocols failed to account for the effect of muscle fatigue on H-reflex responses and this could have potentially led to considerable bias. Finally, we did not include an assessment of the presynaptic inhibition in our study, despite many investigators have recognized presynaptic mechanisms as the main responsible for the spinal adaptation and the resulting changes in spinal excitability (Lagerquist et al. 2012; Aagaard 2018; Grosprêtre et al. 2018). Additional experiments are therefore warranted to analyse potential modifications of the presynaptic inhibition related to the presently observed increase in spinal excitability following NMES superimposed on voluntary contraction.

## Conclusion

The current study findings indicated that NMES superimposed on voluntary isometric contraction of the ankle plantar flexor muscles leads to acute potentiation of the H-reflex response, whereas the H-reflex was inhibited after passive NMES only and did not change after the isometric voluntary contractions only. We suggest that these changes could be due to greater excitation of motor neuronal pools as a result of increased somatosensory stimuli in conjunction with descending supraspinal commands, which synergistically potentiate Ia spinal reflex pathways. The findings of the current study provide novel information about exercise-dependant modulation of spinal excitability, which could have significant implications for the development of specific training and rehabilitation protocols.

## Abbreviations

NMES: Neuromuscular electrical stimulation
ISO: Isometric contraction
MVIC: Maximal voluntary isometric contraction
sEMG: Surface electromyography
SOL: Soleus muscle
TA: Tibialis anterior muscle
ANOVA: Analysis of variance
